# History of a Case of Intussusceptio

**Published:** 1840

**Authors:** John Dawson

**Affiliations:** Green county, Ohio


*February*, 1840.

---

Art. VI.—*History of a Case of Intussusceptio, in which the patient recovered after discharging twenty-nine inches of Intestine.* By Dr. John Dawson, Jr., of Green county, Ohio.

The subject of this case was a boy, J. H. T., 6 years of age, of sanguineous temperament, and predisposed to spasmodic pains in the bowels. He was attacked with these pains and vomiting on Saturday, May 25th, 1839.

I was called to the case the same day and commenced the treatment by fomentations to the epigastric region, a mercurial cathartic, and an anodyne, in the event of a free discharge from the bowels.

Sunday, 8 o'clock, A. M.—Medicine has had no effect on the bowels, vomiting still continues, patient ejects nothing but the secretions of the stomach, pulse 90 to the minute and regular. I now ordered 12 grs. calomel, followed by an infusion of senna.

Monday, 9 o'clock, A. M.—Patient had much restlessness the previous night, medicine not operated yet, vomiting more frequent, accompanied with severe remitting pains in the epigastric, and right and left hypochondriac regions. Injections of jalap and neutral salts were now ordered. Upon their third repetition the obstinate torpor of the bowels gave way and a copious discharge of green crude currants made their appearance in the alvine evacuation.

Tuesday, 7 o'clock, A. M.—Medicine operated three times the previous day and evening, gastric distress not mitigated. Tongue clean and red, skin above the natural temperature, intolerance of warm drinks, pulse somewhat contracted and quick, extremities cool, epigastrium tender. Venesection and a vesicatory to the epigastrium were now resorted to. Also tartaric acid dissolved in a solution of gum acacia, every few hours.

Wednesday.—Evacuations from the stomach lessened in frequency, pulse small and tense, skin hot and dry, tongue covered with undefinable patches of white fur, and granulated on its sides and apex. I now prescribed pills composed of a grain of calomel and a grain of opium, one to be taken every four hours.

Thursday, 4 o'clock, P. M.—Dr. J. Templeton appeared in consultation. Gastric uneasiness moderated, but bowels obstinately constipated. *A tumour about three inches in diameter* now made its appearance in the right lumbar region. With the Doctor's advice vigorous enemata were freely administered and the pills above referred to continued.

Friday, Saturday and Sunday.—No essential change for either better or worse. During this time however there was occasional pain in the bowels, and they continued torpid. The abdomen became distended somewhat, and gas-bubbles could be heard traversing the alimentary canal. On Sunday evening I ordered oleum ricini ℥j., invited to action in two hours by an enema, a blister to the abdomen, and dressed when it took effect with an emollient cataplasm.

Monday, June 3d.—Medicine has taken no effect on the bowels, abdomen much distended and tympanitic, pulse frequent, small and tense, tongue coated white, and the papillæ protruded. I now ordered an emulsion of saccharum solution ℥ss.; oleum terebinthinæ ʒj; oleum ricini ℥iij; and tinct. opii. 30grs. Two drams of this to be administered at intervals of two hours.

Tuesday, 4 o'clock, P. M.—Emulsion produced copious discharges of a pale yellow color, approaching to the natural. With these discharges, contrary to my expectation, the patient's strength was lessened, the action of the heart and arteries became irritated and irregular, abdominal intolerance to pressure increased, the tongue assumed a white glazed appearance with red granulated edges; the countenance contracted and anxious; and excrutiating pain in any other position than on the back with the knees drawn up.

Wednesday, June 5.—Patient better; abdominal pain lessened; the frequency of the pulse and its tenseness giving way; the pale contracted countenance gradually assuming a lively expression, and the abdominal protuberance somewhat retracted.

The patient from this time wore the appearance of convalescence, was dieted carefully, and on the 11th June expelled per anum, with a stool, twenty-nine and a half inches of intestine, including the appendix vermiformis, part of the cecum and colon.

You will observe, 1st—That with the exception of the *tumor* that made its appearance in the right side, this disease wore the appearance of *gastritris,* followed by *peritonitis.* And that the remedies addressed to the case were in accordance with that view of its pathology. Perhaps now, no better diagnostic makes its appearance in intussesception or gastritis than the *tumor* incident to the former.

2d.—That on Tuesday, June 2d, the patient had symptoms of the escape of fæcal matter into the cavity of the abdomen.

3d.—That the expulsion of the diseased bowel took place on the seventh day after the first attack.

